# Plasticity in medaka gonadotropes via cell proliferation and phenotypic conversion

**DOI:** 10.1530/JOE-19-0405

**Published:** 2020-01-23

**Authors:** Romain Fontaine, Eirill Ager-Wick, Kjetil Hodne, Finn-Arne Weltzien

**Affiliations:** 1 Department of Preclinical Sciences and Pathology, Faculty of Veterinary Medicine, Norwegian University of Life Sciences, Oslo, Norway

**Keywords:** estradiol, proliferation, transdifferentiation, puberty

## Abstract

Follicle-stimulating hormone (Fsh) and luteinizing hormone (Lh) produced by the gonadotropes play a major role in control of reproduction. Contrary to mammals and birds, Lh and Fsh are mostly produced by two separate cell types in teleost. Here, we investigated gonadotrope plasticity, using transgenic lines of medaka (*Oryzias latipes*) where DsRed2 and hrGfpII are under the control of the fshb and lhb promotors respectively. We found that Fsh cells appear in the pituitary at 8 dpf, while Lh cells were previously shown to appear at 14 dpf. Similar to Lh cells, Fsh cells show hyperplasia from juvenile to adult stages. Hyperplasia is stimulated by estradiol. Both Fsh and Lh cells show hypertrophy during puberty with similar morphology. They also share similar behavior, using their cellular extensions to make networks. We observed bi-hormonal gonadotropes in juveniles and adults but not in larvae where only mono-hormonal cells are observed, suggesting the existence of phenotypic conversion between Fsh and Lh in later stages. This is demonstrated in cell culture, where some Fsh cells start to produce Lhβ, a phenomenon enhanced by gonadotropin-releasing hormone (Gnrh) stimulation. We have previously shown that medaka Fsh cells lack Gnrh receptors, but here we show that with time in culture, some Fsh cells start responding to Gnrh, while *fshb* mRNA levels are significantly reduced, both suggestive of phenotypic change. All together, these results reveal high plasticity of gonadotropes due to both estradiol-sensitive proliferation and Gnrh promoted phenotypic conversion, and moreover, show that gonadotropes lose part of their identity when kept in cell culture.

## Introduction

Gonadotropes are key players in the control of the reproductive function as part of the brain-pituitary-gonad axis (Harris 1951, Weltzien *et al*. 2004). Located in the anterior part of the pituitary, they produce the two gonadotropins: follicle-stimulating hormone (Fsh) and luteinizing hormone (Lh) (Weltzien *et al*. 2014). Fsh and Lh are mostly produced by the same cell in mammals (Nakane 1970), while the opposite occurs in teleost fish, where Fsh and Lh are produced by two different cell types (Nozaki *et al*. 1990, Schmitz *et al*. 2005, Kanda *et al*. 2011, Weltzien *et al*. 2014). Therefore, teleosts seem ideal models to study the development and the different properties of Fsh and Lh cells, as well as the differential regulation of Fsh and Lh synthesis and release (Yaron *et al*. 2003, Weltzien *et al*. 2014).

However, despite the general understanding of one hormone one cell type in teleosts, several observations have challenged this hypothesis. Indeed, gonadotropes producing both gonadotropins were found in several teleost species (e.g. Mediterranean yellowtail (Hernandez *et al*. 2002), zebrafish, tilapia (Golan *et al*. 2014) and European hake (Candelma *et al*. 2017)). On the other hand, gonadotropes expressing only one hormone were described in mammals (Childs *et al*. 1982, Childs 1983). Previous publications have pointed out the fact that Fsh and Lh share the same developmental basis in fish, similar to what is found in mammals (Weltzien *et al*. 2014) suggesting that Fsh and Lh cells may not be so different from each other in fish, and more similar to the mammalian gonadotropes than we perhaps have anticipated.

Medaka is a powerful teleost model for which several tools have been developed to study its genetics and development (Wittbrodt *et al*. 2002, Shima & Mitani 2004). Recently, our team developed two transgenic lines where (i) hrGfpII reporter protein synthesis is controlled by the endogenous medaka *lhb* promotor using bacterial artificial chromosome (BAC) homologous recombination technology with 103-kb flanking sequence to the *lhb* gene (Hildahl *et al*. 2012) and (ii) DsRed2 synthesis is controlled by the endogenous medaka *fshb* promotor using plasmid construction containing 3833 bp of the fshb promoter sequence (Hodne *et al*. 2019), enabling the study of the gonadotrope cells in more detail.

Previously, several studies conducted on Lh cells in medaka have explored and investigated basic parameters including morphology, ontogeny and regulation of Lh cells. In medaka, Lh cells have been found to participate in the plasticity of the pituitary during puberty through hypertrophy and estrogen-sensitive hyperplasia during puberty (Fontaine *et al*. 2019). Lh cells have also been shown to make neuron-like projections allowing homotypic networks (Grønlien *et al*. 2019), to express gnrh receptors (*gnrhr*), and to respond to gnrh stimuli by increasing their action potential frequency and intracellular calcium concentration (Strandabo *et al*. 2013, Hodne *et al*. 2019). However, very little is known about Fsh cells and if and how they contribute to pituitary plasticity during puberty. Therefore, using the recently developed transgenic lines where Fsh and Lh cells can be identified, we investigated gonadotrope plasticity in the medaka pituitary, examining both proliferation and phenotypic plasticity. In addition, we investigated the presence and the origin of bi-hormonal (expressing both Fsh and Lh) cells in medaka.

## Materials and methods

### Animal maintenance

Wild-type (WT, d-rR strain), transgenic tg(*lhb*-hrGfpII) (Hildahl *et al*. 2012), tg(*fshb*-DsRed2) and double transgenic tg(*lhb*-hrGfpII/*fshb*-DsRed2) (Hodne *et al*. 2019) medaka (*Oryzias latipes*) were maintained at 28°C on a 14/10 h light/dark cycle in a re-circulating system with reverse osmosis dosed-salt water (pH 7.6 and conductivity of 800 µs). Fish were fed three times a day, once with live brine shrimp and twice with dry feed (Gemma; Skretting, Invergordon, UK). Experiments were performed according to the recommendations of the care and welfare of research animals at the Norwegian University of Life Sciences, and under the supervision of authorized investigators. Specifically, the bromodeoxyuridine (BrdU) experiments were approved by the Norwegian Food Safety Authorities (FOTS ID 8596).

### Primary pituitary dispersed cell cultures

Cell cultures were prepared as described in detail in Ager-Wick *et al*. (2018). For measuring the volume of DsRed2 and hrGfp-II expressing cells, four cell cultures were prepared either from 15 adult or due to their smaller size, 25 juvenile pituitaries of tg(*lhb*-hrGfpII/*fshb*-DsRed2) animals from each sex. For quantification of mRNA levels at different time points, cell cultures were prepared by dissociating cells from 25 adult tg(*lhb*-hrGfpII) females. Cells were then plated in three different wells (each corresponding to a different sampling time point) in a 48-well plastic plate (Sarstedt, Germany) coated with poly-L-lysine (Sigma), prepared in a laminar flow hood by adding 50 µL poly-D-lysine, leaving for 1 min before removing the liquid, washing in 500 µL MQ water and leaving the coated wells to dry in UV-light for approximately 30 min.

For investigation of phenotypic conversion, six cell cultures from males and four from females tg(*lhb*-hrGfpII/*fshb*-DsRed2) were prepared. A sub-set of these cell cultures (*n* = 2 from each sex) were treated 4 h after being plated by adding Gnrh1 (concentration 10 µM; H-Glu-His-trp-ser-His-Gly-Leu-Ser-Pro-Gly-OH trifluorocetate salt, >97% purity, Bachem AG, Budendorf, Germany) into the medium, a concentration previously shown to induce calcium and electrophysiological responses in Lh and Fsh cells (Strandabo *et al*. 2013, Ager-Wick *et al*. 2018, Fontaine *et al*. 2018, Hodne *et al*. 2019). Time lapse was recorded as described below for 3 days.

### qPCR

(i) *fshb* mRNA was quantified during development using WT medaka as described in (Hildahl *et al*. 2012). Briefly, a LightCycler 480 Real-Time PCR system (Roche), with SYBR Green (Roche) was used. Pools of synchronized embryos (see Table 2 in Hildahl *et al*. 2012) were collected in RNAlater for RNA isolation and cDNA synthesis. (ii) *gnrhr1b*, *gnrhr2a*, *gnrhr2b*, *lhb* and *fshb* mRNA were quantified from cell cultures at three different time points: 1 h, 24 h and 72 h after plating the dissociated cells. Cells where mechanically detached from the plate by scraping the cells using the pipette in 300 µL of TRIzol and further submitted to phenol-chloroform RNA extraction using GlycoBlue (Invitrogen) as carrier. Experiments were performed in quadruplicate and triplicate respectively, for proper statistical analysis. Using primers previously used and validated by sequencing the amplicons in Hildahl *et al*. (2012), Burow *et al*. (2019) and Hodne *et al*. (2019) (Table 1), we found that the expression of 16s rRNA (*16s*) was the most stable across larval development, and the combination of glyceraldehyde 3-phosphate dehydrogenase (*gapdh*), ribosomal protein L7 (*rpl7*) and 18s rRNA (*18s*) was the most stable across time in cell culture according to BestKeeper software (Pfaffl *et al*. 2004), and thus used to normalize the expression analysis, using an efficiency-corrected relative quantification method (Weltzien *et al*. 2005).

**Table 1:** List of primers used for qPCR.

Gene name	Forward sequence 5′–3′	Reverse sequence 5′–3′	Amplicon length	qPCR efficiency
*16s*	CGATCAACGGACCGAGTTACC	AATAGCGGCTGCACCATTAGG	119	2.05
*rpl-7*	TGCTTTGGTGGAGAAAGCTC	TGGCAGGCTTGAAGTTCTTT	98	2.03
*β-actin*	ACCCTGTCCTGCTCACTGAA	GCAGGGCTGTTGAAAGTCTC	92	2.07
*fshb*	GACGGTGCTACCATGAGGAT	TCCCCACTGCAGATCTTTTC	73	2.03
*lhb*	CCACTGCCTTACCAAGGACC	AGGAAGCTCAAATGTCTTGTAG	100	2.00
*gnrhr1b*	TCCTGCTACACATCCACCAG	GCCTTTGGGATGATGTCTGT	88	1.99
*gnrhr2a*	GGGCGATGAGTGTGATCCTC	CCCGAGTGGCACATTGAGT	96	1.99
*gnrhr2b*	TTGAGATATCAAGCCGCATC	GAGTCCTCATCCGAGCTTTG	99	2.02
*18s*	CCTGCGGCTTAATTTGACTC	AACTAAGAACGGCCATGCAC	118	2.02
*gapdh*	CCTCCATCTTTGATGCTGGT	ACGGTTGCTGTAGCCAAACT	75	2.01

### Steroid treatments and BrdU incubation

To study effects of sexual steroids on Fsh and Lh cell proliferation, three groups of tg(*lhb*-hrGfpII) adult fish (six females and six males) were incubated for 6 days in daily renewed system water containing 100 μg/L of either 17β-estradiol, testosterone or 11-ketotestosterone (Sigma; diluted in 96% ethanol), similar to what has been used in (Thompson 2000, El-Alfy & Schlenk 2002), and shown to significantly increase estradiol plasma level after 7 days of treatment (Thompson 2000). Control fish (six of each sex) were incubated for 6 days with ethanol only (diluted 1:10^5^ in water). The experiment was repeated once. Immediately after steroid treatment, the fish were treated with 1 mM BrdU (Sigma) diluted in water with 15% DMSO for 8 h. Fish were then killed, brain and pituitary were collected and fixed in 4% paraformaldehyde overnight, and gradually dehydrated and stored in 100% methanol until use.

### Immunofluorescence

To investigate cell proliferation, tissues were labelled for BrdU, PCNA as well as for Fshβ with immunofluorescence (IF), as previously described (Fontaine *et al*. 2013, Burow *et al*. 2019, Fontaine *et al*. 2019). Briefly, IF was performed on free-floating sections obtained after the tissues were included in 3% agarose and parasagittaly sectioned (60 μm) with a vibratom (Leica). Because the fluorescence of the endogenous DsRed2 is quenched with the epitope retrieval treatments required for BrdU and PCNA staining, tg(*lhb*-hrGfpII) animals were used and IF for Fshβ, with a custom-made polyclonal rabbit anti-medakaFshβ (1:500 (Burow *et al*. 2019)) was performed. Nuclei were stained with DAPI (1:1000; 4′,6-diamidino-2-phenylindole dihydrochloride; Sigma).

To investigate Lhβ protein production in cell culture, we prepared three cell cultures from female tg(*fshb*-DsRed2) and three cell cultures from female tg(*lhb*-hrGfpII/*fshb*-DsRed2). Cells were treated with Gnrh1 as described earlier. To investigate whether Fsh cells possess Lhβ protein right after dissociation, one cell culture from tg(*fshb*-DsRed2) line was fixed 1 h after dissociation. The other cell cultures were incubated 72 h with GnRH. Cells were fixed with 4% paraformaldehyde (diluted in phosphate saline buffer, PBS) for 15 min at room temperature (RT) and washed with PBS. Then, cells were incubated 15 min with 0.3% triton (Sigma) diluted in PBS and washed again with PBS. Cells were then incubated in blocking (10% normal goat serum diluted in PBS) before being incubated with the previously validated antibody anti rabbit anti-medakaLhβ (1:1000 in blocking solution (Burow *et al*. 2019)) overnight at 4°C. As control, one of the three cell culture was not receiving the primary antibody. Colour revelation was performed by washing the cells with PBS and then incubating them 1 h at RT with a secondary antibody conjugated to either Alexa-488 for tg(*fshb*-DsRed2) cell cultures or Alexa-647 for tg(*lhb*-hrGfpII/*fshb*-DsRed2) cell cultures (Invitrogen).

### Fluorescence *in situ* hybridization (FISH)

FISH was performed as described in Fontaine *et al*. (2013). Briefly, six adult fish from each sex from the tg(*lhb*-hrGfpII) line were killed with ice cold water, and brain and pituitary were collected and fixed in 4% paraformaldehyde. Tissues were then gradually dehydrated and stored in 100% methanol until use. Tissue were then rehydrated and sectioned parasagittally (60 μm) with a vibratome (Leica). Free-floating sections were hybridized with *Cyp19a1b*-digoxigenin-tagged (DIG) and *fshb*-fluorescein-tagged (FITC) riboprobes (Okubo *et al*. 2011, Fontaine *et al*. 2019) for 18 h at 55°C. Colour revelation was performed with sheep anti-DIG, and anti-FITC conjugated with peroxidase (1:250; Roche), together with TAMRA-conjugated and Cy5-conjugated tyramides for FISH (Roche). Nuclei were stained with DAPI (1:1000; Sigma).

### Imaging

For imaging of the tg(*lhb*-hrGfpII/*fshb*-DsRed2) line during ontogeny (8–10 unsexed fish per stage) or for investigating the presence of bi-hormonal cells (12 unsexed fish per stage), no treatments where needed and endogenous hrGfpII together with DsRed2 were directly visualized. For all, vibratome slices were mounted between slide and coverslip with antifade mounting medium Vectashield (Vector, UK), and spacers were added between the slice and the coverslip when mounting whole pituitaries. Time-lapse recordings of dissociated pituitary cells were performed in a humid chamber at 26°C with 1% CO_2_ (Ager-Wick *et al*. 2018). All confocal images were acquired using a LSM710 microscope (Zeiss) with 10×, 25×, 40× or 63× (respectively N.A. 0.3, 0.8, 1.2 or 1.4) objectives. Channels were acquired sequentially to avoid signal crossover between filters. Z-projections from confocal image stacks were obtained using Fiji software (v2.0.0 (Schindelin *et al*. 2012)). 3D reconstruction was built using 3D-viewer plugin (Schmid *et al*. 2010).

### Calcium imaging and Gnrh1 stimulation

Calcium imaging and Gnrh1 stimulation were performed as described in (Hodne *et al*. 2019). Briefly, a total of three dishes of dissociated adult female tg(*fshb*-DsRed2) pituitary cells were used. Following 3 days in culture, the cells were gently washed in artificial BSA-free extracellular solution (ECS: in mM: NaCl 134, KCl 2.9, MgCl_2_ 1.2, HEPES 10, and glucose 4.5, pH 7.75 and 290 mosmol), then incubated in 5 µM Fluo4-AM dye (ThermoFisher Scientific) for 30 min before incubation in ECS added 0.1% BSA for 20 min. In total, 29 cells were stimulated with Gnrh1 (10 µM dissolved in ECS with 0.1% BSA) using puff ejection (20 kPa through a 2 MΩ glass pipette, 30–40 µm from the target cell). Cells were imaged using a sCMOS camera (optiMOS, QImaging, British Columbia, Canada) with exposure time 50 to 80 ms and sampling frequency 0.5 Hz using µManager software, v1.4 (Edelstein *et al*. 2014). Relative fluorescence intensity was calculated after background subtraction as changes in fluorescence (F) divided by the average intensity of the first 15 frames (F0). Data analysis was performed using Fiji software.

### Counting and measurements

Counting of Fsh cells was performed blindly using Cell Profiler software (v2.1.0 (Carpenter *et al*. 2006)) as described in Fontaine *et al*. (2019), from eight to nine animals from each sex and stage. Double-labeled cells (BrdU/hrGfpII or BrdU/DsRed2) after steroid and BrdU treatments were manually counted using Fiji software and cell-counter plugin. Cell volume was measured by recording Z-stacks of dissociated cells a few minutes after being plated and using Fiji software and the voxel counter plugin for 9–36 cells per group. The fluorescence intensity in the mean region of interest (ROI) was measured with Fiji on five different cells from two different cell cultures using 10× objective. For good clarity of the figure only three cells were kept.

### Statistics

Data were analyzed using GraphPad Prism (v8.0), tested for normality using the Shapiro–Wilk normality test. Significant difference between groups was set at *P* < 0.05, and non-parametric tests were used when data were not normally distributed. See figure legends for details.

## Results

### Ontogeny of Fsh cells in the pituitary

**Figure 1:**
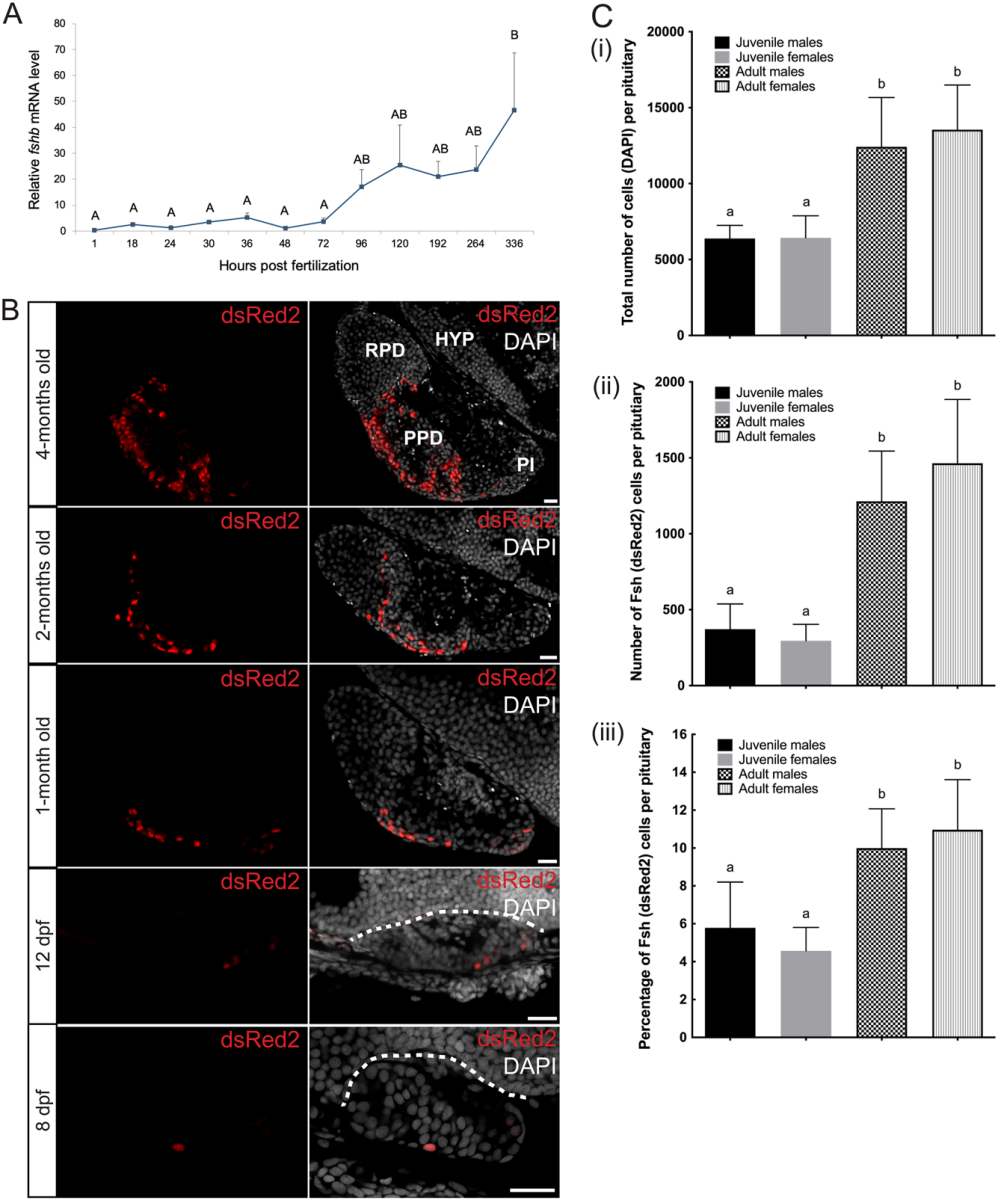
(A) Relative *fshb* mRNA levels during early development in pooled medaka larvae by quantitative polymerase chain reaction (qPCR) analysis. *fshb* gene expression was normalized to *16s* gene expression using an efficiency adjusted relative quantification method. Data are presented as mean relative expression + s.e.m., *n* = 4. Data were tested for normal distribution with the Shapiro–Wilk normality test, and relative mRNA levels were significantly different (*P* < 0.05) using one-way ANOVA followed by a Tukey–Kramer HSD *post hoc* analysis when letters are different (A and B). (B) Ontogeny of DsRed2 producing cells in the tg(*fshb*-DsRed2) line. Parasagittal sections of the brain and the pituitary for fish from 1-month old up to 4-months old, and of the whole embryo for younger stages, without (left panels) or with nuclear (DAPI) staining (right panels). Dotted lines delimit the dorsal part of the pituitary. Scale bars: 20 μm. (C) Cell counting for the four different groups of fish: juvenile males (*n* = 9) and females (*n* = 9), and adult males (*n* = 8) and females (*n* = 9). (i) Mean (+s.d.) of the total number of cells in the pituitary. (ii) Mean (+s.d.) of the number of DsRed2 cells in the pituitary. (iii) Mean (+s.d.) of the percentage of DsRed2 cells related to the total number of cells in the pituitary. For each graph, data were tested for normal distribution with the Shapiro–Wilk normality test, and one-way ANOVA with Tukey’s multiple comparison test revealed significant differences (*P* < 0.05) when letters are different (a and b).

qPCR (Fig. 1A) shows that relative expression of *fshb* mRNA in the embryo starts to increase after 72 h post fertilization (hpf; 3 days). It becomes significantly different from the early time points after 336 hpf (14 days). To investigate at which time the first Fsh cells appear, we looked at the endogenous DsRed2 (Fig. 1B) fluorescence starting with adult fish, back to younger stages in the tg(*fshb*-DsRed2) line. First, we did not observe any DsRed2 cells outside of the pituitary at all studied stages. Second, we found the first cells to arise around 8 days post fertilization (dpf), with a single DsRed2 cell observed in two of eight studied embryos at this stage. Third, we observed an increasing number of DsRed2 between each studied stage along development.

Therefore, we counted the number of DsRed2 cells in the pituitary as well as the total number of cells using the nuclear DAPI staining, and calculated the percentage of DsRed2 cells in the pituitary (Fig. 1C), in juveniles (2-month old) and adults (6-month old) in both sex. While the number of cells in the pituitary increased significantly between juvenile and adult stages, there was no significant differences between sex at any stage. The same observation was made for the number of DsRed2 cells and the percentage of DsRed2 cells in the pituitary. In adults however, there was a noticeable tendency for higher numbers of cells and DsRed2-positive cells in females as compared to in males.

### Proliferation of Fsh cells

**Figure 2:**
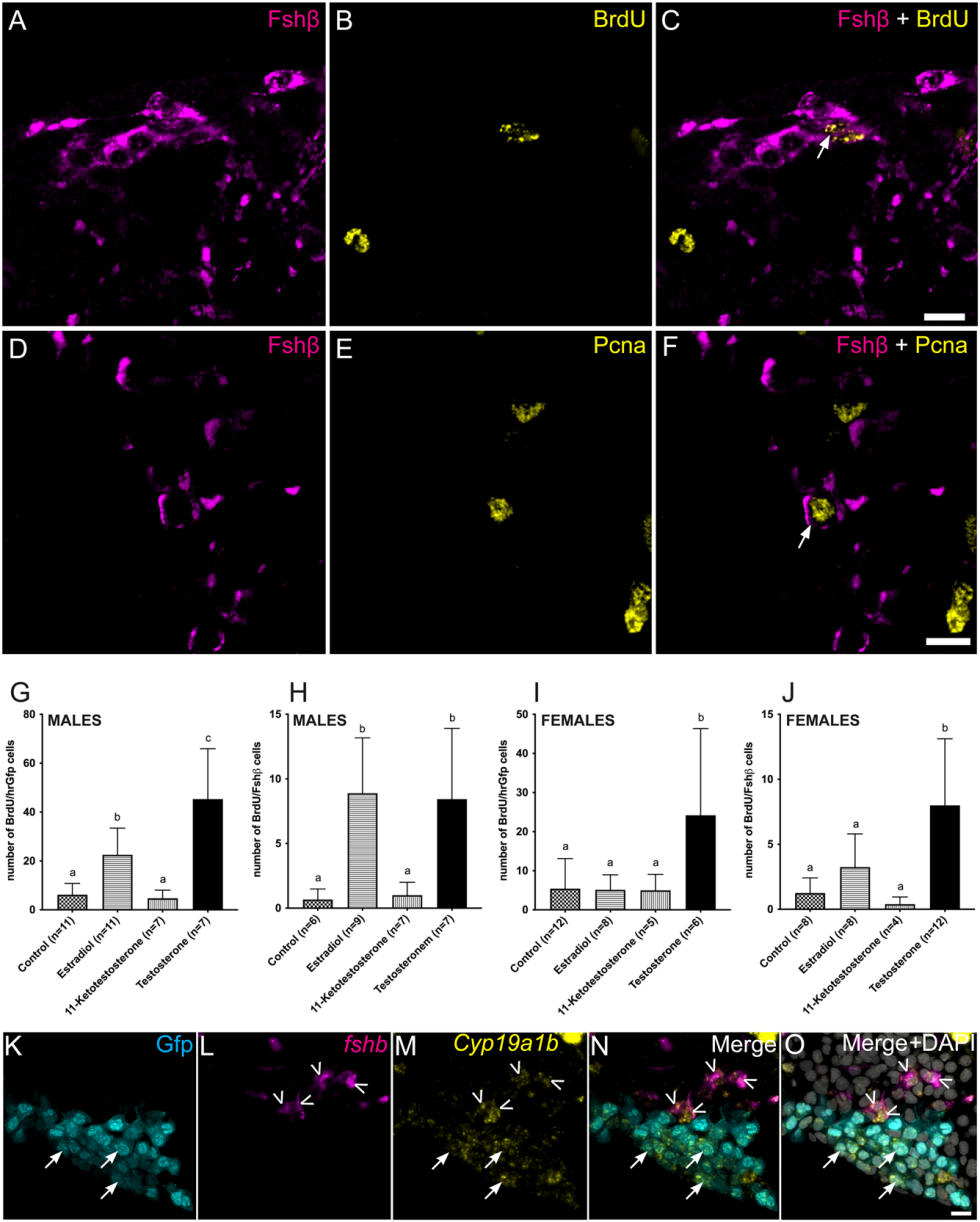
(A, B and C) Confocal plan images of a parasagittal section from a tg(*lhb*-hrGfpII) adult female medaka pituitary incubated in BrdU for 8 h and labeled by immunofluorescence for Fshβ (magenta) and BrdU (yellow). (D, E and F) Confocal plane images of a parasagittal section from an adult WT female medaka pituitary labeled by immunofluorescence for Fshβ (magenta) and proliferating cell nuclear antigen (PCNA; yellow). Scale bars: 10 μm. (G, H, I and J) Graphics presenting the mean (+s.d.) number of double labelled cells, BrdU/hrGfpII, using BrdU immunofluorescence and endogenous hrGfpII fluorescence (G and I) or BrdU/Fshβ using bi-color Fshβ and BrdU immunofluorescence (H and J) in the pituitary from adult medaka males (G and H) and females (I and J). Fish were treated for 8 h in BrdU after 6 days treatment in either estradiol, 11-ketotestosterone, testosterone or ethanol (control). ‘*n*’ represents the number of individual fish analyzed. Data were found to not follow a normal distribution according to the Shapiro–Wilk normality test, thus non-parametric test was used. For each graph, Kruskal–Wallis test followed by Dunn’s multiple comparison test revealed significant differences (*P* < 0.05) when letters are different (a, b and c). (K, L, M, N and O) Multi-color fluorescent *in situ* hybridization for *fshb* and aromatase (*cyp19a1b*) with nuclear staining (DAPI) in the tg(*lhb*-hrGfpII) adult female pituitary. Arrows show the Lh (Gfp) cells, and arrowheads the *fshb*-expressing cells which also express *cyp19a1b*. Scale bar: 10 μm.

We then looked for the origin of the new DsRed2-positive cells. IF for proliferating cell nuclear antigen (PCNA) together with Fshβ showed some cells expressing both proteins (Fig. 2A, B and C). In addition, IF for BrdU together with Fshβ on fish incubated for 8 h in BrdU solution revealed that some Fshβ-producing cells had integrated BrdU, therefore confirming active cell division (Fig. 2D, E and F).

We then investigated the effect of sex steroids on gonadotrope cell proliferation. Unfortunately, some of the treated fish were not Gfp positive and some HCL treatment to unmask Fshβ epitope did not work, leading to reduced number of samples in some of the groups (see ‘*n*’ in Fig. 2G, H, I and J). Nevertheless, steroid treatments before BrdU incubation and labelling by IF revealed that, contrary to 11-ketotestosterone (11-KT), both estradiol (E2) and testosterone were able to significantly increase the number of both BrdU/hrGfpII and BrdU/Fshβ cells in male pituitaries compared to control. In females, testosterone was able to increase the number of BrdU/Fshβ and BrdU/hrGfpII cells compared to control, although the difference was only significant for BrdU/Fshβ. In contrast, other treatments did not affect the number of BrdU/hrGfpII or BrdU/Fshβ cells in females.

Finally, we investigated the expression of aromatase (Cyp19a1b) in *fshb*-expressing cells and Gfp cells using multicolor FISH in both sexes. We observed in both males and females without noticeable differences, the expression of *Cyp19a1b* in both Lh (arrows) and Fsh (arrowheads, Fig. 2K, L, M, N and O).

### Distribution of Lh and Fsh cells in the pituitary

**Figure 3:**
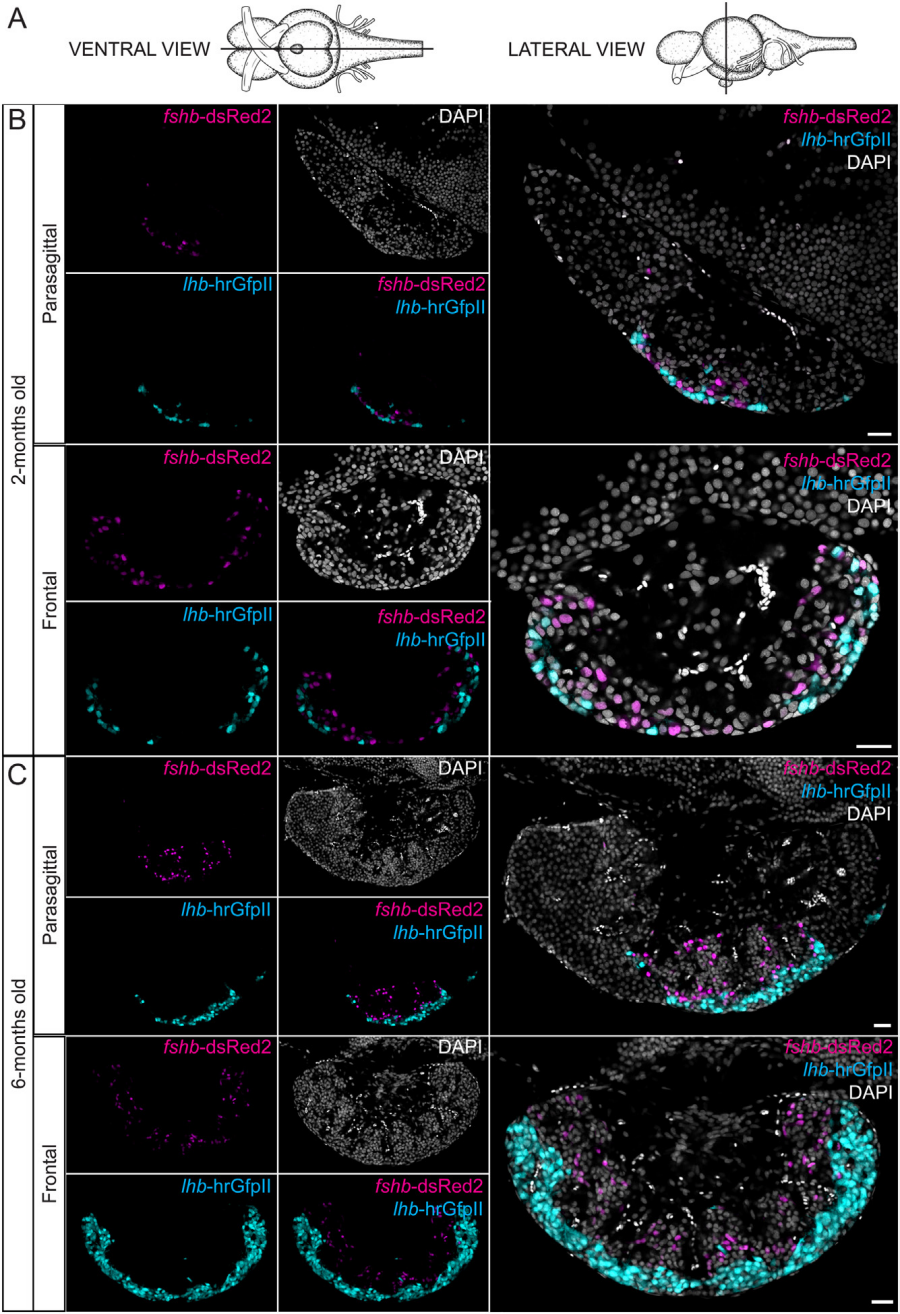
(A) Schemas presenting the position of the sections made in the brain and pituitary used for the following images, from the ventral and ventral point of view providing respectively parasagittal and frontal sections. (B) Confocal plan images of the endogenous fluorescence from 2-months old and 6-months old females tg(*lhb*-hrGfpII/*fshb*-DsRed2) medaka brain and pituitary, in parasagittal and frontal sections. Sections are shown without or with nuclear (DAPI) staining. Scale bars: 20 μm.

Based on observations in the double transgenic line (*lhb*-hrGfpII/*fshb*-DsRed2), hrGfpII and DsRed2-positive cells are distributed in the median part of the pituitary in adult fish (Fig. 3). This becomes even more clear when looking at the distribution in a 3D reconstituted image of the pituitary (Juveniles: Supplementary Movies 1 and 2; adults: Supplementary Movies 3 and 4, see section on supplementary materials given at the end of this article). We did not observe any difference between sex (data in males not shown); however, we could clearly see that in adults, hrGfpII cells are situated along the ventral and lateral surface of the pituitary while DsRed2 are located more internally. While hrGfpII cells to a large extent are clustered, DsRed2 cells seem more individualized and spread out. In juveniles, some of the DsRed2 cells were closer to the surface, some even touching the ventral and lateral surface of the pituitary, while this was never observed in adults where Lh cells cover the entire ventral and lateral surface.

**Figure 4:**
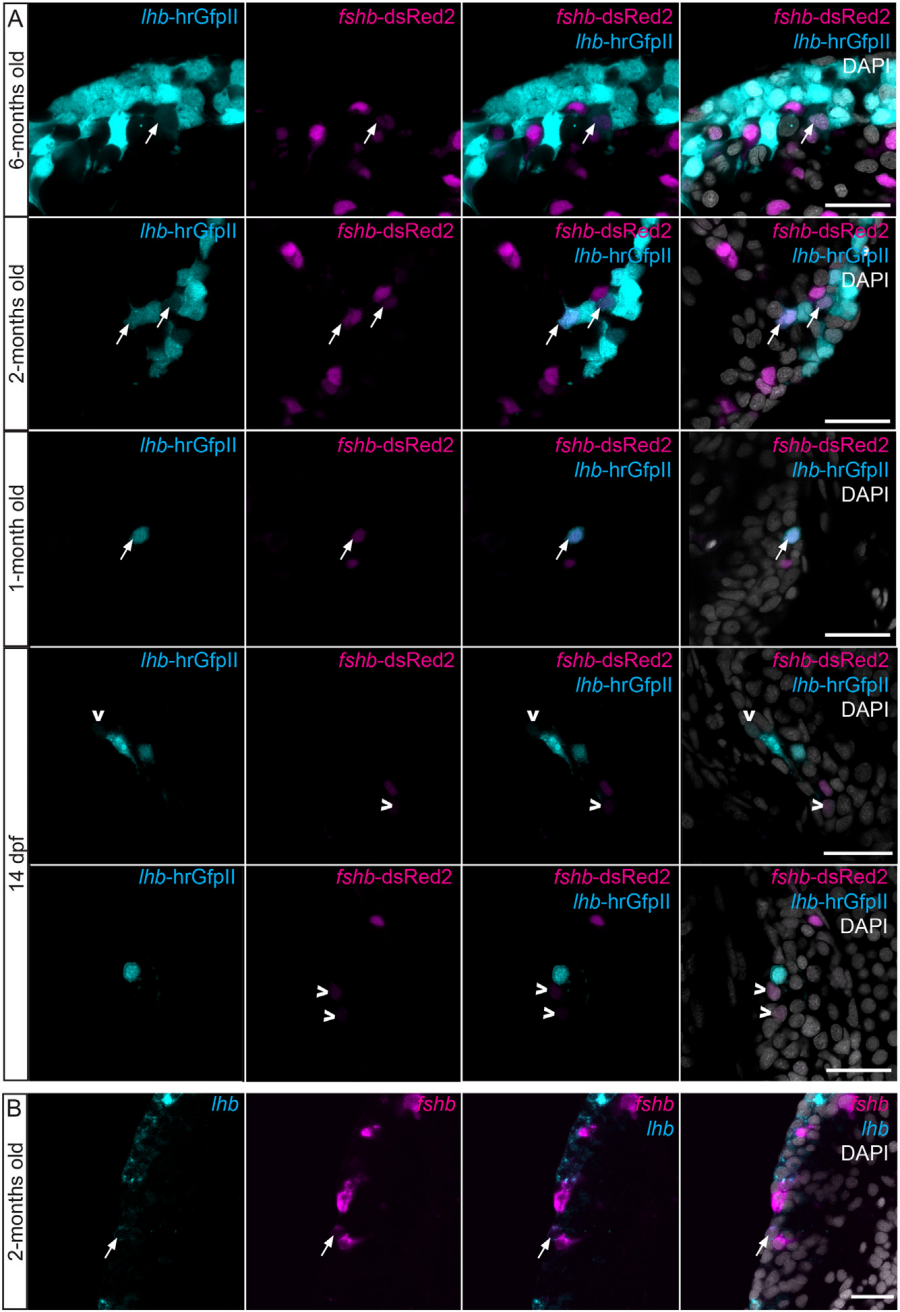
(A) Confocal plan images from the endogenous fluorescence in parasagittal sections from 6-months old, 2-months old, 1 month old and 14 dpf unsexed tg(*lhb*-hrGfpII/*fshb*-DsRed2) medaka brain and pituitary. (B) Confocal plan images of a parasagittal section from the brain and pituitary of a 2-months old WT fish labeled by multi-color FISH for *lhb* and *fshb* mRNA. Cells expressing both hrGfpII and DsRed2 (A) or *lhb* and *fshb* (B) are shown with white arrows while cells showing weak expression of DsRed2 or hrGfpII are shown with white arrow heads (A). Scale bars: 20 μm.

Interestingly, a few cells were positive for both hrGfpII and DsRed2 in 6-, 2- and 1-month-old fish (Fig. 4A). Such co-expression was also shown in WT animals using FISH for *lhb* and *fshb* mRNA (Fig. 4B). However, cells expressing both reporter proteins were never observed in 14 dpf larvae (*n* = 12 larvae), at which time the first Lh cells arise in the pituitary. At this developmental stage, a few cells were weakly labelled either hrGfpII or DsRed2, but never in the same cell (Fig. 4A).

### Morphology of Fsh and Lh cells

**Figure 5:**
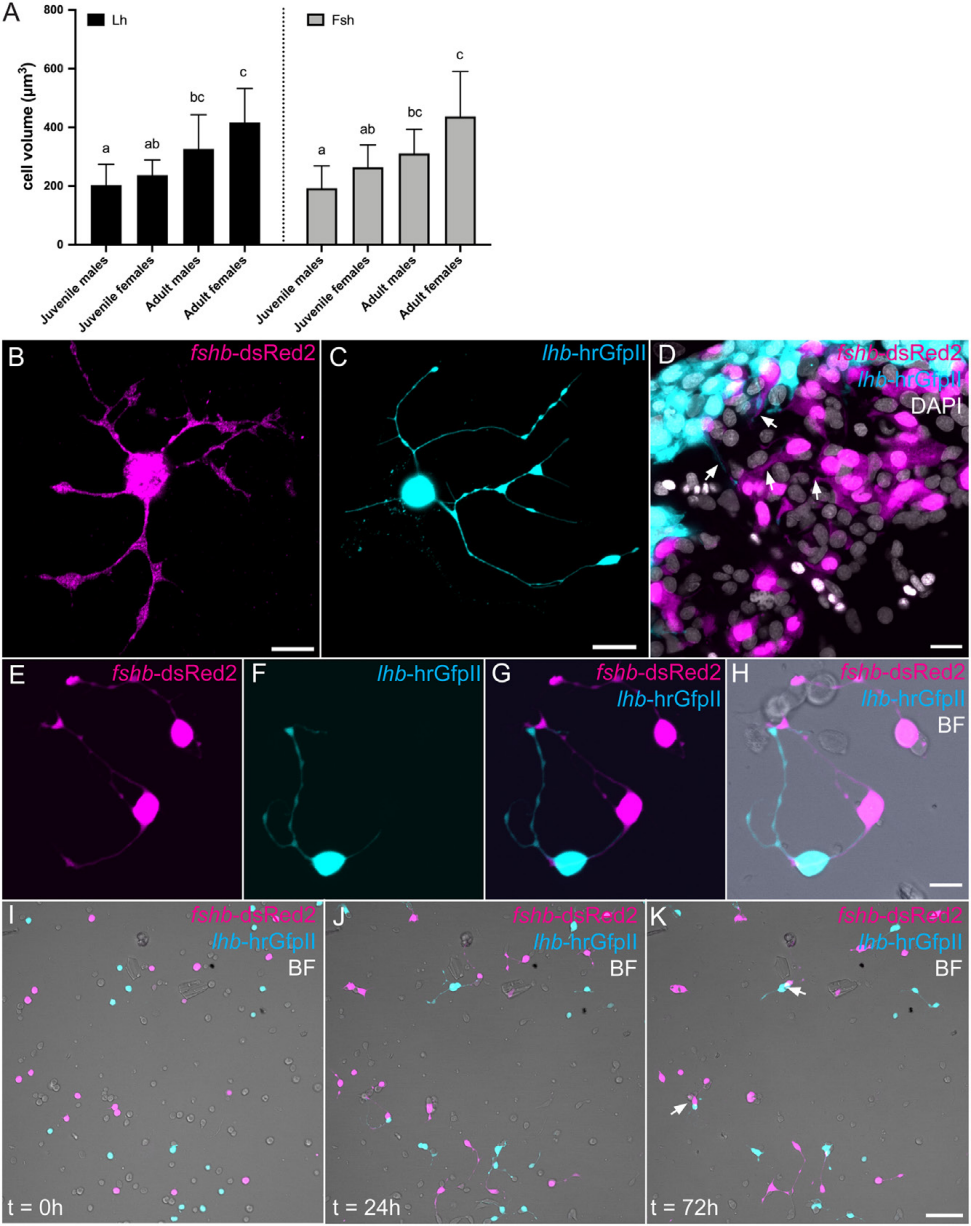
(A) Graphic showing the calculated cell volume of Lh (hrGfpII) or Fsh (DsRed2) cells in cell culture from tg(*lhb*-hrGfpII/*fshb*-DsRed2) animals, just after cells were dissociated and plated. Cell volume was measured in cells from juvenile males (*n* = 10 cells) and females (*n* = 9 cells) as well as in adult males (*n* = 13 cells) and females (*n* = 23 cells). Data were log-transformed and tested for normal distribution with the Shapiro–Wilk normality test. Two-way ANOVA with Tukey’s multiple comparison test revealed significant differences (*P* < 0.05) when letters are different (a, b, and c). (B and C) Confocal plan image from a dsRed positive (Fsh) and hrGfpII (Lh) cell respectively, in cell culture for 24 h. (D) Confocal plan image from a parasagittal section of a pituitary from adult tg(*lhb*-hrGfpII/*fshb*-DsRed2) female with nuclear (DAPI) staining. Arrows show the extensions of the cells in the tissue. (E, F, G and H) Confocal plan images from pituitary cell culture from tg(*lhb*-hrGfpII/*fshb*-DsRed2) adult females, 24 h after dissociation showing heterotypic network between a dsRed positive (Fsh) and hrGfpII (Lh) cell as well as other unknown cell types revealed by the brightfield (BF) image. Scale bars: 10 μm. (I, J and K) Time lapse image of a pituitary cell culture from tg(*lhb*-hrGfpII/*fshb*-DsRed2) adult females showing clustering of dsRed-positive (Fsh) and hrGfpII (Lh) cells as shown by the arrows. Scale bar: 50 μm.

Using the double transgenic line (*lhb*-hrGfpII/*fshb*-DsRed2), we investigated cell morphology. Measuring the volume of both hrGfpII and DsRed2-positive cells in dissociated pituitary cell cultures from juveniles and adults (Fig. 5A), we observed a volume increase from juvenile to adult stages in both cell types. Interestingly, the cell volume is similar for hrGfpII and DsRed2-positive cells at both analyzed life stages.

In addition, we observed that both in cell culture as well as in fixed tissue slices, hrGfpII and DsRed2-positive cells show seemingly similar long extensions from the cell body (Fig. 5B, C and D). In dissociated cell culture, they use these extensions to make connections between them (homotypic and heterotypic networks; Fig. 5E, F, G, H and Supplementary Movie 5). They also use these extensions for clustering (Fig. 5I, J, K and Supplementary Movie 5).

### Phenotypic conversion of Fsh cells into Lh cells in medaka primary pituitary cell culture

**Figure 6:**
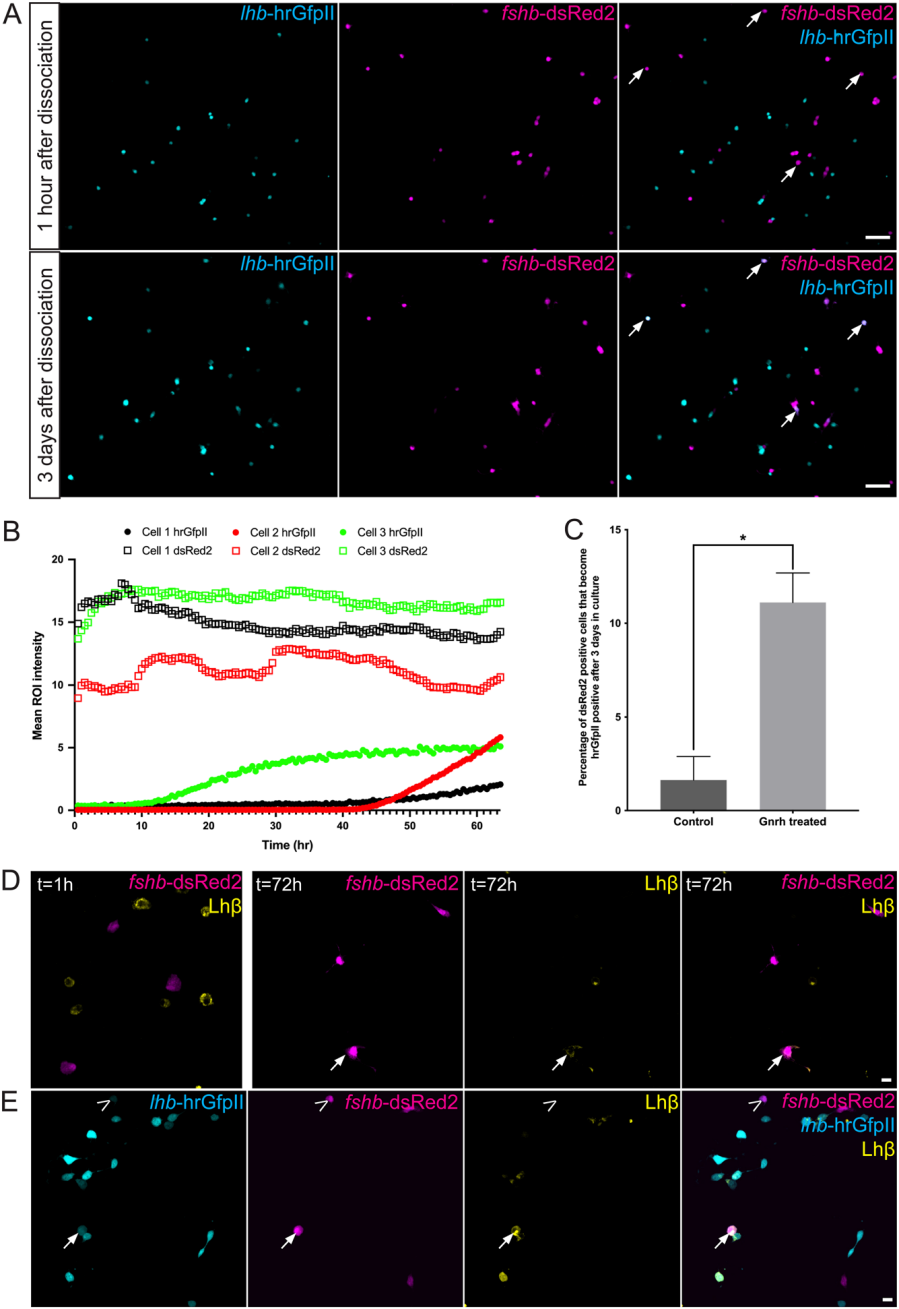
(A) Confocal plan images of a pituitary cell culture from tg(*lhb*-hrGfpII/*fshb*-DsRed2) adult males 1 hour (top panels) and 3 days (bottom panels) after dissociation. Arrows show DsRed2-positive cells that are becoming hrGfpII-positive cells during the 3 days. (B) Graphic presenting the mean fluorescent ROI intensity for hrGfpII and DsRed2 from three different cells over time, from two different cell cultures imaged with a 10× objective. (C) Graphic showing the mean (+s.e.m.) of the percentage of DsRed2-positive cells that have started to produce hrGfpII after 3 days in cell culture with Gnrh1 (*n* = 4 cell cultures from two males and two females) or without (control *n* = 6 cell cultures from four males and two females). Cell cultures from different sexes were pooled as they presented similar results for each treatment. Data were found to not follow a normal distribution according to the Shapiro–Wilk normality test, thus the non-parametric Mann–Whitney test was used to investigate significant difference in the proportion of Fsh (DsRed2) cells changing phenotype with or without Gnrh1 stimulation. (D) Confocal plan on cell cultures from adult tg(*fshb*-DsRed2) females, 1 or 72 h after dissociation and incubation with Gnrh1, and labelled for Lhβ by immunofluorescence. The arrow shows a DsRed2-positice cell labeled for Lhβ. (E) Confocal plan on cell cultures from adult tg(*lhb*-hrGfpII/*fshb*-DsRed2) females, incubated with Gnrh1 for 3 days and labelled for Lhβ by immunofluorescence. The arrow shows a cell producing both Gfp and DsRed2 and labeled for Lhβ while the arrowhead shows another cell producing both Gfp and DsRed2 but not labeled for Lhβ. Scale bars: 10 μm.

Recording time lapse images of dissociated primary pituitary cell culture from the double transgenic line (*lhb*-hrGfpII/*fshb*-DsRed2), we observed that some cells which were not expressing hrGfpII at the beginning were able to start to produce it with time (Fig. 6A, B and Supplementary Movie 6). Most of the cells starting to express hrGfpII in the culture where DsRed2 positive and some of them start to produce hrGfpII already after 15 h in cell culture. In addition, while we observed an increase of hrGfpII fluorescence over time, we did not observe any decrease of DsRed2 fluorescence in these cells (Fig. 6B). We also found that adding Gnrh1 in the medium, significantly increased the number of DsRed2 positive cells starting to produce hrGfpII (Fig. 6C). Interestingly, we also observed cells that initially were not labelled starting to express hrGfpII, but we never observed any hrGfpII expressing cell starting to produce DsRed2.

Then, we performed IF for Lhβ to investigate whether the Fsh (DsRed2-positive) cells observed to become Gfp-positive in culture where also producing the Lhβ protein. First, without the use of the primary antibody, we could not observe any labelling. This confirms the specificity of the secondary antibodies used in cell culture. Second, using the tg(*fshb*-DsRed2) line, we could not observe any DsRed2-positive cells labelled for Lhβ 1 h after dissociation, while several could be observed after 72 h incubation with Gnrh1 (Fig. 6D). Third, with the double transgenic line (*lhb*-hrGfpII/*fshb*-DsRed2) we found that some cells, both Gfp- and DsRed2-positives, were labelled for Lhβ while we could not observe any DsRed2-only cell labelled for Lhβ (Fig. 6E).

### Activity of Fsh cells upon Gnrh stimulation in medaka primary pituitary cell culture

**Figure 7:**
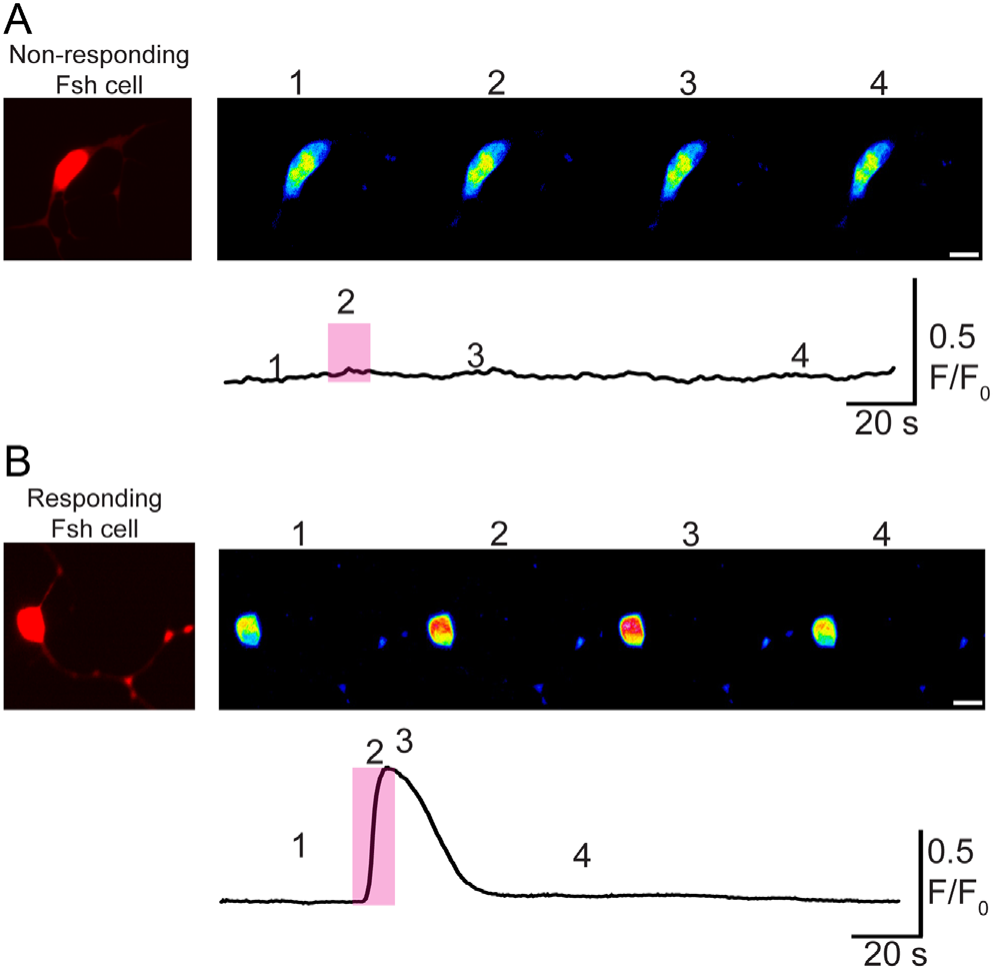
Cytosolic calcium measurements in Fsh cells following 1 µM Gnrh1 stimulation using 3 days cultivated dissociated pituitary cells from adult female tg(*fshb*-DsRed2) medaka. In total 16 of 29 Fsh cells (55%) responded to Gnrh1. Recording of the fluorescence intensity after stimulation with Gnrh1 in a (A) non-responding Fsh cell and (B) responding Fsh cell. (A and B) Upper micrographs represent four images from a time lapse of an Fsh cell following Gnrh stimulation (pink shaded rectangle). Below, the corresponding trace were each number (1–4) represents the timepoints of the selected pictures above. Scale bars on images: 10 μm.

In our previous study of adult female medaka, we demonstrated that Fsh cells lack Gnrh receptors in tissue sections and do not show calcium or electrophysiological responses upon Gnrh stimulation when investigating the cells shortly after dissociation (Hodne *et al*. 2019). Using dissociated primary pituitary cell cultures from adult female tg(*fshb*:DsRed2) line and the calcium imaging technique, we observed that 3 days after plating, 55% of the DsRed2 expressing cells show a transient elevation in cytosolic calcium with a latency of 2–5 s upon Gnrh1 stimulation (Fig. 7). The response usually lasted between 20 and 60 s before returning to baseline values.

### Temporal gene expression in primary medaka pituitary cell culture

**Figure 8:**
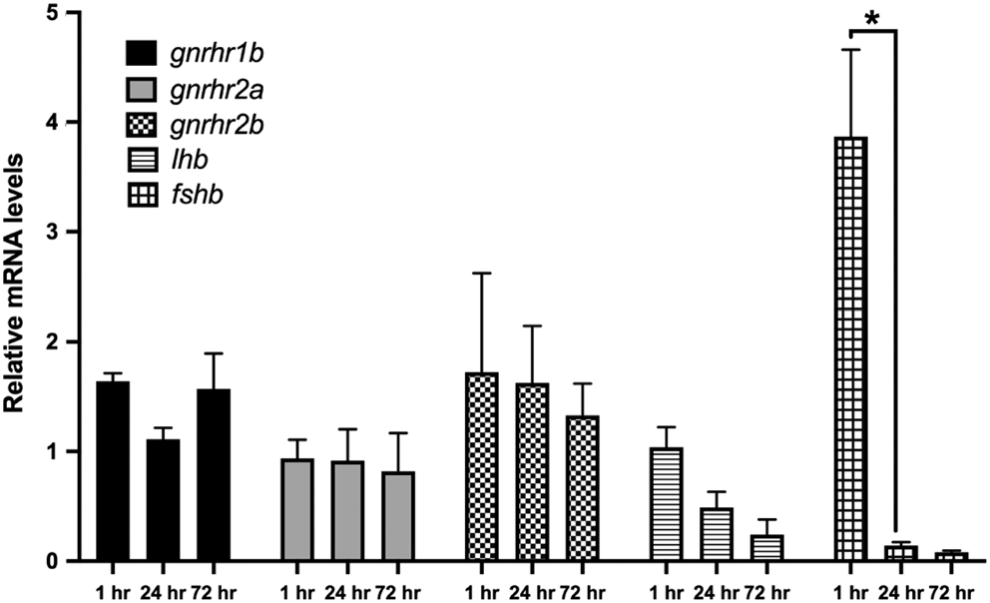
Temporal relative mRNA levels for *lhb*, *fshb*, *gnrhr1b*, *gnrhr2a* and *gnrhr2b* in cell culture from tg(*lhb*-hrGfpII) adult female pituitaries. The mRNA levels of the genes of interest were reported to the level of a combination of reference genes including *rpl7*, *gapdh* and *18s* RNA. Data were tested for normal distribution with the Shapiro–Wilk normality test, and two-way ANOVA with Tukey’s multiple comparison test revealed significant differences (* when *P* < 0.05).

We analyzed gene expression over time in cell culture of *lhb*, *fshb* and the three Gnrhr found in the medaka pituitary (*gnrhr1b*, *gnrhr2a* and *gnrhr2b*) according to (Hodne *et al*. 2019). Three time points were studied (Fig. 8), 1 h, 24 h and 72 h after the dissociated cells were plated. We observed a significant reduction in *fshb* expression already after 24 h. In contrast, no significant change in expression was observed for *lhb*, *gnrhr1b*, *gnrhr2a* and *gnrhr2b* over time.

## Discussion

Lh and Fsh are key players in the BPG axis, controlling reproductive function. While medaka Lh cells have been well described (Hildahl *et al*. 2012, Fontaine *et al*. 2019), little is known about Fsh cells and the population they form in the medaka pituitary. In general, less is known about Fsh cells than for Lh cells in teleost fish. In this study, we used the recently developed and validated medaka transgenic lines allowing for the visualization and localization of Fsh-producing (DsRed2) and Lh-producing (hrGfp) cells (Hodne *et al*. 2019), referred to as Lh and Fsh cells, following the definition of endocrine cells used by Pogoda & Hammerschmidt (2007).

We first studied the ontogeny of Fsh cells and demonstrated that while a significant increase of the *fshb* mRNA relative amount cannot be observed before 14 dpf, the first Fsh cell can already be observed in the pituitary after 8 dpf. This is prior to the observation of the first pituitary Lh cells which arise at 14 dpf (Hildahl *et al*. 2012). This is comparable to zebrafish, where Fsh arise before Lh cells (4 and 28 dpf for Fsh and Lh cells, respectively (Golan *et al*. 2014)). Contrary to what has been previously described for Lh cells (Hildahl *et al*. 2012), we did not observe any Fsh cell outside of the pituitary in medaka. We then observed that similar to in Lh cells (Fontaine *et al*. 2019), the number of Fsh cells as well as the percentage of cells they represent in the pituitary increase between juvenile and adult stages. In addition, we demonstrate that the cell volume is also increasing between juvenile and adult stages, which is in agreement with the previous observation where Lh cell size was also observed to increase. Therefore, both the proportion and the cell volume of gonadotropes (Lh and Fsh) are increasing in the medaka pituitary between juveniles and adults in both sex, certainly because reproduction plays a more important role in adults. These observations are similar to in mammals where an increasing number and size of gonadotropes has been observed during diestrus (Childs 1986, 1995). Interestingly, we noticed that the proportion of Lh cells is higher than for Fsh, in both juveniles (approximately 11 and 6%, respectively) and adults (approximately 13 and 10%, respectively).

Three hypotheses can explain the increasing number of gonadotropes in the pituitary. First, the division and differentiation of some progenitor cells. Second, the division of the gonadotrope themselves, and third, a phenotypic conversion of some of the differentiated pituitary cells. While the first hypothesis seems to have a primary role in mammalian pituitary plasticity (Florio 2011) and cannot be ruled out as some multipotent progenitor cells have been described previously in the dorsal part of the medaka pituitary (Fontaine *et al*. 2019), we focused our work on the two last hypotheses.

Proliferation has previously been described for Lh cells in the medaka pituitary, and here we demonstrate that this is also the case for Fsh cells. PCNA, an essential protein for DNA replication during the cell cycle, and BrdU which has been demonstrated to be a useful and reliable marker for labelling recently divided and currently dividing cells (Bauer & Patterson 2005), were both observed in Fsh cells, confirming active cell division. Division of hormone producing cells is not restricted to fish as this has also been observed in the mammalian pituitary (Kominami *et al*. 2003) including gonadotropes themselves (Childs & Unabia 2001).

Sex steroids play crucial roles in multiple systems related to reproduction, and E2 has been shown to play an essential role in medaka reproduction (Kayo *et al*. 2019). While the number of Fsh cells as well as Lh cells labelled by BrdU increased after E2 or testosterone treatment in males, this was not the case following treatment with 11-KT (a non-aromatizable androgen). These results therefore suggest that E2, and testosterone after aromatization into E2, are able to promote both Fsh and Lh cell proliferation in male medaka. In females however, only testosterone was able to increase the proliferation of Fsh cells. These results are in agreement with our previous study where we observed a stimulatory effect of E2 on Lh cell proliferation in males but not in females (Fontaine *et al*. 2019), perhaps due to higher endogenous levels of E2 in females (Bhatta *et al*. 2012, Kayo *et al*. 2020). Several studies have addressed the role of E2 and aromatizable androgens on the activity of Lh and Fsh cells in both mammals (Nett *et al*. 2002) and fish (Yaron *et al*. 2003, Zohar *et al*. 2010). In mammals, some studies have reported a negative effects of steroids on gonadotrope cell proliferation: Mitotic gonadotropes drastically increase after castration in male rats (Sakuma *et al*. 1984), and ovariectomy in female rats (Smith & Keefer 1982). In fish, almost nothing is known about the role of steroids on gonadotrope cell proliferation. On gonadotrope cell activity, the effect of steroids has been shown to diverge depending on the species and the physiological state of the fish. Both positive and negative feedback have been observed. E2 and androgen treatments have been shown to inhibit gonadotrope activity in Indian catfish (Sundararaj & Goswami 1968), goldfish (Billard & Peter 1977), and rainbow trout (Billard & Reinaud 1978) pituitary. But other studies have reported positive effects of E2 and aromatizable androgens on gonadotrope cells, such as in Atlantic salmon (Crim & Peter 1978), European eel (Olivereau & Olivereau 1979) and rainbow trout (Crim *et al*. 1981). Indeed, testosterone and E2 have previously been shown to enhance Gnrh-stimulated Lh release in Rainbow trout (Crim & Evans 1983), goldfish (Trudeau *et al*. 1991), Atlantic croaker (Khan *et al*. 1999) and Black Porgy (Yen *et al*. 2002). Trudeau and colleagues hypothesized that the positive action of sex steroids on Gnrh responsiveness was due to an increase in pituitary Gnrh receptor number, although they could not demonstrate effect on pituitary Gnrh affinity or receptor number, *in vitro* (Trudeau *et al*. 1993). In medaka, we describe a positive effect of E2 and testosterone, on the proliferation of both gonadotrope cell types, therefore suggesting that testosterone and E2 may have an additional role on gonadotrope cells by controlling their proliferation. Whether testosterone and E2 enhance Gnrh-stimulated Lh release in medaka and if this effect involves the proliferation of gonadotrope cells remain to be investigated.

In this study, we used relatively high doses of steroids (100 µg/L). These doses have previously been used in medaka (El-Alfy & Schlenk 2002, Fontaine *et al*. 2019) and shown to be the lowest dose that significantly increase E2 plasma levels after 7 days of treatment (Thompson 2000). Interestingly, a recent study tested different techniques for steroid administration and showed that lower concentrations of E2 can be used to increase E2 plasma level in medaka and that medaka seems to bioconcentrate E2 in the blood (Kayo *et al*. 2020). However, the authors investigated the plasma level of E2 for only 24 h after E2 treatment, thus further experiments need to be done, with different exposure times and concentrations, to clearly identify the role of steroids on the different components of the BPG axis including gonadotrope cell proliferation. Also, while the vehicle (ethanol) used in this study was highly diluted (1:10^5^) as compared to previous studies (1:10^2^–1:10^4^) discussed in von Krogh *et al*. (2019), whether the vehicle can have an effect on pituitary cell proliferation remains to be investigated. Finally, we confirm the results observed in Fontaine *et al*. (2019) and show that some Lh cells express aromatase. In addition, we found that some Fsh cells also express aromatase suggesting that they may participate in the control of their own proliferation.

To test the third hypothesis about phenotypic plasticity we used the double transgenic line. We observed some gonadotropes labelled by both hrGfpII and DsRed2 in adult and juvenile stages suggesting that some cells could express both gonadotropic hormones in the medaka pituitary. We then confirmed that some cells were expressing both *lhb* and *fshb* mRNA using two colour FISH technique. Dual phenotype has been reported in other teleost fish, including the Mediterranean yellowtail (Hernandez *et al*. 2002), European hake (Candelma *et al*. 2017), zebrafish and tilapia (Golan *et al*. 2014). It is presently unknown whether these cells are progenitor cells in a transient phenotype of differentiation towards one hormone phenotype, or fully differentiated gonadotropes in a transient form during the phenotypic conversion from one hormone phenotype to another or simply with permanent bi-hormonal phenotype. Lh and Fsh cells have been shown to share the same developmental path (Weltzien *et al*. 2014), and the presence of Fsh cells has been revealed in the ventral surface of the pituitary in larval and juvenile stages, in close proximity to the Lh cells. However, we never observed dual labelling in pituitary cells of 14 dpf old larvae, the time when the first Lh cells arise. Instead, we could observe some weakly labelled hrGfpII or DsRed2 cells, suggesting that new gonadotropes arise as monohormonal cells. Therefore, the dual phenotype gonadotropes is probably not expressed in differentiating progenitor cells, but more likely in cells that change phenotype at a later stage.

We found that Lh and Fsh cells are similar in morphology. They have similar volume in juveniles and in adults and show, both *in vivo* and *in vitro*, extensions allowing networking as previously shown for Lh (Grønlien *et al*. 2019). Here, we show that Lh and Fsh cells show similar behaviour as they connect and cluster in cell culture using these extensions. While these similarities suggest a similar genetic background, which has already been shown (Weltzien *et al*. 2014), they would also make it easy for a phenotypic conversion between the two phenotypes. We previously reported that some cells from unknown identity where able to start to produce Lh with time in cell culture (Fontaine *et al*. 2019). Here, we demonstrated that in cell culture, some Fsh cells can change phenotype and start to produce *lhb*, and that Gnrh stimulates this phenotypic conversion. Interestingly, we did not observe any obvious decrease of DsRed2 fluorescence in the Fsh cells suggesting that the Fsh cells may become bi-hormonal, but fluorescent reporter proteins have usually relatively long half-life (about 24–30 h in mammalian cells) (Corish & Tyler-Smith 1999). In addition, we observed that levels of *fshb* mRNA were drastically reduced after 24 h in cell culture, but we cannot identify which cells are responsible for this decrease. It is therefore impossible to determine if the cells that start to produce *lhb* become Lh-monohormonal or bi-hormonal cells. As the tg(*lhb*:hrGfp) line is BAC-based it should widely include all possible enhancer regions of the *lhb* gene suggesting that the control observed on hrGfpII expression indeed occurs for *lhb* expression. This was further supported by our observation of some hrGfpII/DsRed2 cells found to contain Lhβ. Indeed, we observed that while none of the Fsh cells were producing Lhβ after dissociation, several Fsh cells were found labelled for Lhβ after 3 days in cell culture with Gnrh1. In addition, these DsRed2/Lhβ cells were shown to be also hrGfpII-positive using the double transgenic line. It should be noted that we never observed Lh cells becoming Fsh positive. These results are similar to the one observed *in vitro* in rats (Childs 1985) where mono-hormonal Fsh cells have been found to become bi-hormonal when stimulated with Gnrh. This phenotypic conversion of Fsh cells has also been described *in vivo* in the Rhesus Monkey during sexual maturation (Meeran *et al*. 2003). In addition, it has already been described in rats (Denef *et al*. 1978) and sheep (Taragnat *et al*. 1998), that Gnrh was responsible for a change in the pituitary gonadotrope population by regulating the existence of LH-monohormonal, FSH-monohormonal and bi-hormonal gonadotrope subtypes. Whether this phenomenon is reversible and if other compounds could have similar or opposite effects, remains to be tested. These experiments also need to be performed *ex vivo* to confirm that in medaka, phenotypic plasticity is not just due to cell culture conditions.

**Figure 9:**
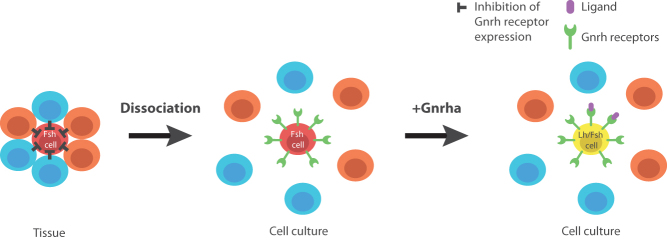
Schematic drawing of the working hypothesis. After dissociation, Fsh cells seem to start to express Gnrh receptors while they do not *in vivo* according to Hodne *et al*. (2019). Therefore, an unknown factor is preventing the expression of Gnrh receptors by Fsh cells *in vivo*. After dissociation, this factor is removed and some Fsh cells become able to respond to Gnrh stimuli. Application of Gnrh is then able to promote the expression of *lhb* in the Fsh cells that express Gnrh receptors and have become Gnrh sensitive.

It is also interesting to notice that while Gnrh promotes phenotypic conversion and thus increases the number of double labelled cells *in vitro*, a lower number of double labelled cells were observed *in vivo* (where gonadotropes could receive Gnrh input) than *in vitro*. These observations suggest that something prevents the phenotypic conversion of Fsh cells by Gnrh *in vivo*. It has recently been shown that in adult female medaka, *in vivo* Fsh cells do not possess any *gnrhr*, and do not respond (electrically nor by changes in cytosolic Ca^2+^ levels) to Gnrh stimuli after dissociation and maintained for a short period in culture (less than 48 h (Hodne *et al*. 2019)). Here, we show that after 3 days in cell culture, a subset of Fsh cells (about 50%) start responding to Gnrh stimuli by increasing the intracellular calcium concentration. This suggests that Fsh cells are changing phenotypic characteristics after being cultivated for an extended time without close contact with other cells and without brain and feedback inputs, may start to produce gnrhr. However, we did not observe any increase of *gnrhr* expression in medaka pituitary cell culture. This may be due to the relatively low number of Fsh cells in our cultures or/and the relatively high expression of *gnrhr* in the other pituitary cell types, thus hiding small increases of expression by Fsh cells. Indeed, a study in cod primary pituitary cell culture has reported an increase of the gene expression levels between day 2 and day 7 of one Gnrh-receptor (Gnrhr2a) found to be expressed in gonadotropes in this species (Hodne *et al*. 2012). Together, these observations suggest that Fsh cells need an unknown input to maintain their Fsh-only phenotype by preventing Gnrh-receptor expression in Fsh cells *in vivo*, such as described in Fig. 9. Further studies are needed to identify the factors playing a role in the maintenance of Fsh status and its origin (brain, gonads or pituitary itself), but the recent study from Hodne *et al*. 2019 showing that gonadotropes form hetero- and homotypic networks makes the hypothesis of an important paracrine factor loss by the dissociation process highly relevant. These observations further support that precautions should be taken about the conclusions when investigating dissociated primary pituitary cell cultures over time.

Many groups have reported a direct stimulation of Fsh cells by Gnrh in cell culture. While we observed that some Fsh cells start to produce *lhb* after only 15 hours in medaka cell culture, most *in vitro* studies use the cells several days after they were dissociated and plated: more than 3 days for coho salmon (Dickey & Swanson 2000), 5 days for rainbow trout (Vacher *et al*. 2000), 2 days for masu salmon (Ando *et al*. 2004), and more than 2 days for Atlantic cod (Hodne *et al*. 2013). In addition, other studies have shown an effect of Gnrh on Fsh cells in more complete systems (pituitary slices or whole pituitary), where cells are kept in a more intact environment and connections with neighbouring cells are preserved (tilapia (Aizen *et al*. 2007) and medaka (Karigo *et al*. 2014)), but a recent study showed that Fsh cells can be activated indirectly through heterotypic pituitary cell networks in medaka (Hodne *et al*. 2019). Therefore, whether Gnrh directly affects Fsh cells in fish should be reinvestigated taking these new findings into account.

To conclude, this study demonstrates that gonadotropes, Lh and Fsh cells, show high plasticity by exhibiting hypertrophy and hyperplasia between juvenile and adult stages. They both proliferate in the medaka pituitary upon estradiol and aromatizable androgen stimulation, and may participate in the control of their own proliferation as they both express aromatase. Fsh cells have the capacity to change their phenotype by starting to produce Lh, and this phenomenon is promoted by Gnrh, and seemingly prevented by an unknown factor *in vivo*. This may explain the low number of gonadotropes observed as bi-hormonal in different fish species. Combined, these two phenomena may participate in adapting hormone production to hormone demand, which differs across the life span of an animal. It also reveals that fish gonadotropes are more similar to the mammalian gonadotropes than we have anticipated.

## Supplementary Material

Supplemental movie 1: 3D reconstruction of whole pituitary from tg(lhb-hrGfpII/fshb-DsRed2) juvenile female medaka imaged by LSM710 confocal with 40X oil objective and built with 3D-viewer plugin (Fiji software). Lh cells (hrGfp-II) are cyan and Fsh cells (DsRed2) are magenta. Anterior to the top.

Supplemental movie 2: 3D reconstruction of whole pituitary from tg(lhb-hrGfpII/fshb-DsRed2) juvenile female medaka imaged by LSM710 confocal with 40X oil objective and built with 3D-viewer plugin (Fiji software). Lh cells (hrGfp-II) are cyan and Fsh cells (DsRed2) are magenta. Nuclei stained with DAPI are in grey.

Supplemental movie 3: 3D reconstruction of whole pituitary from tg(lhb-hrGfpII/fshb-DsRed2) adult female medaka imaged by LSM710 confocal with 25X oil objective and built with 3D-viewer plugin (Fiji software). Lh cells (hrGfp-II) are cyan and Fsh cells (DsRed2) are magenta. Anterior to the top.

Supplemental movie 4: 3D reconstruction of whole pituitary from tg(lhb-hrGfpII/fshb-DsRed2) adult female medaka imaged by LSM710 confocal with 25X oil objective and built with 3D-viewer plugin (Fiji software). Lh cells (hrGfp-II) are cyan and Fsh cells (DsRed2) are magenta. Nuclei stained with DAPI are in grey. Anterior to the top.

Supplemental movie 5: Confocal time-lapse recording of primary pituitary cell culture from tg(lhb-hrGfpII/fshb-DsRed2) adult male showing gonadotropes making extensions and clustering. Imaged with a LSM710 confocal and 40X oil objective in time lapse with 15 min between each picture, from 1 h after the cells have been dissociated and plated and for 72h. Lh cells (hrGfp-II) are green and Fsh cells (DsRed2) are red.

Supplemental movie 6: Confocal time-lapse recording of primary pituitary cell culture from tg(lhb-hrGfpII/fshb-DsRed2) adult male treated with Gnrh1 showing red (DsRed2) cells becoming yellow (starting to produce hrGfp-II). Imaged with a LSM710 confocal and 40X oil objective in time lapse with 15 min between each picture, from 4 h after the cells have been dissociated and plated and for 72h. Lh cells (hrGfp-II) are green and Fsh cells (DsRed2) are red.

## Declaration of interest

The authors declare that there is no conflict of interest that could be perceived as prejudicing the impartiality of the research reported.

## Funding

This work was funded by the Norwegian University of Life Sciences and by the Research Council of Norway, grant numbers 244461 and 243811 (Aquaculture program) and 248828 (Digital Life Norway program).

## Ethics approval

Animal experiments were performed according to the recommendations of the care and welfare of research animals at the Norwegian University of Life Sciences, with specific approval from the Norwegian Food Safety Authority (FOTS ID 8596).

## Author contribution statement

R F, E A W, K H made the experiments. R F, E A W, K H and F A W conceived the research and analyzed the data. R F wrote the manuscript, with input from the other authors.
